# Use of laboratory test results in patient management by clinicians in Malawi

**DOI:** 10.4102/ajlm.v4i1.277

**Published:** 2015-11-18

**Authors:** Kundai Moyo, Carol Porter, Ben Chilima, Reuben Mwenda, Mark Kabue, Lutho Zungu, Abdoulaye Sarr

**Affiliations:** 1 Howard University, Technical Assistance Project, Lilongwe, Malawi; 2 Preventive Health Services, Ministry of Health, Lilongwe, Malawi; 3 Health Technical Support Services (Diagnostics), Lilongwe, Malawi; 4 Senior Laboratory Advisor, Centers for Disease Control and Prevention, Lilongwe, Malawi

## Abstract

**Background:**

Malawi has a high burden of infectious disease. The expansion of programmes targeting these diseases requires a strong laboratory infrastructure to support both diagnosis and treatment.

**Objectives:**

To assess the use of laboratory test results in patient management and to determine the requirements for improving laboratory services.

**Methods**: A cross-sectional study was conducted in 2012 to survey practising clinicians. Two hospitals were purposively selected for observations of clinicians ordering laboratory tests. Twelve management-level key informants were interviewed. Descriptive statistics were conducted.

**Results:**

A total of 242 clinicians were identified and 216 (89%) were interviewed. Of these, 189 (87%) reported doubting laboratory test results at some point. Clinicians most often doubted the quality of haematology (67%), followed by malaria (53%) and CD4 (22%) test results. A total of 151 (70%) clinicians reported using laboratory tests results in patient management. Use of laboratory test results at all times in patient management varied by the type of health facility (*P* < 0.001). Ninety-one percent of clinicians reported that laboratories required infrastructure improvement. During 97 observations of clinicians’ use of laboratory test results, 80 tests were ordered, and 73 (91%) of these were used in patient management. Key informants reported that the quality of laboratory services was good and useful, but that services were often unavailable.

**Conclusion:**

Gaps in the public laboratory system were evident. Key recommendations to enhance the use of laboratory test results in patient management were to strengthen the supply chain, reduce turn-around times, improve the test menu and improve the laboratory infrastructure.

## Introduction

Malawi has a population of 15 million people^1^ and the major disease burden is caused by HIV, malaria and tuberculosis. According to the 2010 Malawi Demographic and Health Survey,^2^ HIV prevalence was 11% amongst adults aged 15–49 years. Almost six million malaria cases are reported annually and contribute to 40% of hospitalisations in children aged five years or younger and 34% of outpatient cases across all age groups.^3^ In the Malaria Indicator Survey conducted in 2012, the prevalence of malaria diagnosed by slide microscopy was 28% nationally.^3^ To address these challenges, Malawi set up the Essential Health Package (EHP), which was designed to reach citizens at all levels and was focused on these high burden diseases. The EHP provides free health services at the point of delivery in an effort to ensure the accessibility of healthcare to all, including the poor.^4^ According to the World Health Organization (WHO), EHPs are set up to ensure that limited resources are concentrated on interventions that provide the best value to achieve efficiency, equity, political empowerment, accountability and effectiveness.^5^

Malawi's Essential Medical Laboratory Services form an integral part of the EHP for providing basic laboratory services. The Medical Laboratory Policy^6^ states in part that ‘the profile of essential laboratory tests shall be standardized and provided for at each level of care and based on the level of support required for the EHP, public health importance, clinical importance, cost and affordability, suitability to the working environment and the level of expertise’. In order to ensure the effectiveness and efficiency of the EHP, resources are directed toward proper diagnosis and management of diseases through either eradication or reduction in prevalence, whilst at the same time ensuring equitable access to health services.

In this article, ‘patient management’ refers to the interaction between clinicians and patients. Clinicians often request laboratory tests as part of the decision-making process, expecting the results to provide answers to the condition of a particular patient for proper management.^7^ According to Wians,^8^ clinicians can request a laboratory test for the following reasons: ruling a disease in or out, monitoring of therapy, screening for congenital diseases and researching the pathology of a disease. A study by Sturm^9^ showed that use of laboratory tests as the basis for prescribing antimicrobial drugs had better patient outcomes compared with basing the diagnostic decision on clinical presentation only.

However, some clinicians often do not use laboratory tests. In an observational study conducted at Ntcheu District Hospital in Malawi, laboratory tests were requested for only 68% of cases that required them and only 73% – 79% of the laboratory results were used appropriately. This means that laboratory tests are not requested for all cases requiring one, and, for tests that are requested, not all influence patient management.^10^ In Kenya, a study was conducted to investigate reasons why clinicians failed to use laboratory test results in patient management. The most common reasons were lack of time for clinicians to order tests, lack of trust in the test results and long turn-around times for receiving tests results.^11^ A similar study conducted in Ghana identified the following potential barriers for using laboratory test results: limited availability of laboratory services, lack of quality of testing, challenges in the delivery of results to clinicians, lack of time for clinicians to order tests, and negative attitudes and behaviours toward laboratory services.^12^ It is important that healthcare providers ensure optimal use of resources for the benefit of their clients. Therefore, determining and addressing the factors that deter clinicians from using laboratory tests for patient management will ensure that laboratory services are improved to meet clinicians’ expectations and enable clinicians to use laboratory tests in patient management more effectively.

The Malawi Ministry of Health (MoH) and its partners are committed to strengthening laboratory services through a five-year national laboratory strategy. Coverage and utilisation of laboratory services in patient management has not been assessed in Malawi on a large scale. This study investigates the factors associated with the use of laboratory test results by clinicians working in public health facilities in Malawi. The study also aimed to determine the requirements for improving laboratory services to enhance usage of laboratory test results in patient management.

## Methods

### Study population

This cross-sectional study collected primary data from practising clinicians across all 75 of Malawi's public hospitals with laboratory facilities from October to November 2012. A clinician was defined as a medical doctor, clinical officer, or medical assistant who was authorised to request laboratory tests. The key informants targeted in this survey were MoH programme managers from the departments of Clinical Services, Nursing, HIV and AIDS, as well as representatives from MoH partners (Clinton Health Access Initiative [CHAI], the Christian Health Association of Malawi [CHAM] and the Laboratory Capacity Consortium [LCC]).

### Sampling

All four of Malawi's central hospitals were included in the study. In each region, the district or CHAM hospitals were selected by stratified systematic sampling. All of the district hospitals in the region were listed in alphabetical order and 30% of the total number of hospitals with laboratories were systematically selected. This was repeated for the CHAM facilities. Fifty clinicians from each of the central hospitals and 10 from each of the district and CHAM facilities were asked to participate. The following 22 sites were selected:

- All central hospitals from each of the three regions (*n* = 4).
- 30% of district hospitals in each region (*n* = 9).
- 30% of CHAM hospitals in each region (*n* = 9).

A list of key informants was purposively compiled from laboratory managers, laboratory partners and programme managers from four MoH departments (HIV, Tuberculosis, Malaria and Clinical Services). All key informants were asked to participate in the interviews by providing their perceptions of laboratory services and the utilisation of laboratory tests in public hospitals. The results of this survey were used to validate the findings of the clinician survey.

### Data collection

Data were collected from clinicians using a questionnaire, which was administered through face-to-face interviews by trained research assistants. The questionnaire solicited demographic information (age, facility, qualifications and years of experience) and information on factors that influence a clinician's decision to request and use laboratory test results. The study focused on the following tests: CD4, glucose, haemoglobin (Hb) or full blood count (FBC), malaria microscopy, tuberculosis microscopy, bacteriology microscopy, culture and sensitivity. An interview guide that contained closed- and open-ended questions was used during the key informant interviews.

Observations of clinicians’ use of laboratory services were conducted at Malamulo Mission and Mzimba District hospitals. A data collection form adapted from Mepham et al.^10^ was used. Without interfering with routine decision-making processes, an independent researcher, who was a qualified practising clinician recruited as a research assistant, observed clinicians conducting consultations with patients and healthcare workers for at least one week in the wards and outpatient departments at the two facilities. For each test ordered, the clinician-researcher noted whether the correct laboratory tests were requested; whether the appropriate specimens were collected and processed by the laboratory; and whether the test result(s) were used in patient management.

### Data analyses

Themes were identified for qualitative data and analysed initially using descriptive statistics. The chi-square test was conducted to determine factors associated with use of laboratory tests. P-values less than 0.05 were considered to be statistically significant. All analyses were conducted using STATA™ version 11.0 (StataCorp, College Station, Texas, United States).

### Ethical considerations

Ethical clearance for this study was granted by the National Health Sciences Research Committee (NHSRC) of Malawi in June 2011 and the US Centers for Disease Control and Prevention, Center for Global Health, Office of the Associate Director for Science/Laboratory Science in August 2012 as an activity not including human subjects research. After the purpose of the study, procedures, risks and benefits and participants’ rights were explained, all participants were asked for written consent before a questionnaire was administered. Oral consent to observe consultations was obtained from both the clinician and patient for the clinic observations. Names of participants and other personal identifiers were not documented; unlinked and anonymous codes were used on the questionnaires.

## Results

A total of 242 potentially eligible clinicians were targeted for interviews based on the most current list of practising clinicians at the 22 health facilities selected in 2012. Of the 242 clinicians targeted, 216 (89%) were interviewed, 21 (9%) refused to participate and five (2%) were unavailable.

### Demographic characteristics

Table 1 summarises the demographic characteristics of the clinicians who participated. Men comprised 167 of the 216 respondents (77%). The mean age of respondents was 32 years and the modal age category was the 25–29 age-group, comprising 68 (31%) respondents. The median years of service as a clinician was four (interquartile range: 2–8). The distribution of the type of institutions where the respondents were working was as follows: central/referral hospitals (*n* = 100; 46%), district hospitals (*n* = 88; 41%) and CHAM hospitals (*n* = 28; 13%). A majority of respondents were clinical officers (*n* = 132; 61%), 21% (*n* = 45) were medical assistants, 10% (*n* = 21) were medical doctors and 8% (*n* = 17) were in other categories that included nurses and specialist clinicians. The majority (*n* = 202; 94%) of the clinicians were trained in Malawi, and about one-third (*n* = 76; 36%) had been in the work-force for less than three years.

**TABLE 1:** Characteristics of clinicians in Malawi reporting use of laboratory test results, October–November 2012.

Variables	Characteristic (N = 216)	n (%)
Sex	Female	44 (21)
	Male	167 (77)
	Not indicated	5 (2)
Age	20–24 years	35 (16)
	25–29 years	68 (31)
	30–34 years	43 (20)
	35–39 years	25 (12)
	≥ 40 years	45 (21)
Number of years practicing as a clinician	≤ 3 years	76 (36)
	≥ 3–5 years	43 (20)
	≥ 5–10 years	45 (21)
	≥ 10–15 years	18 (9)
	≥ 15–20 years	10 (5)
	≥ 20 years	18 (9)
Professional title	Medical doctor	21 (10)
	Clinical officer	132 (61)
	Medical assistant	45 (21)
	Other	17 (8)
Country of training	Malawi	202 (94)
	Other	13 (6)
Institution	Central hospital	100 (46)
	District hospital	88 (41)
	CHAM hospital	28 (13)

CHAM, Christian Health Association of Malawi.

### Patterns of ordering and use of laboratory tests

Table 2 describes clinicians’ ordering and use of laboratory results and their levels of confidence in the results. A majority (*n* = 151; 70%) of respondents indicated that all tests ordered were used for patient management, whilst 30% (*n* = 64) indicated that tests results were used sometimes. Most of the 216 respondents (*n* = 189; 87%) reported the occasional questioning of laboratory results for any specific reason and only a small proportion (*n* = 23; 11%) had never questioned laboratory results. Respondents indicated that they would request a repeat test if they doubted the initial laboratory results. The results of Hb/FBC tests were the most commonly doubted, with 67% (*n* = 140) of clinicians reporting they would request a repeat test. Approximately half requested repeat malaria testing (*n* = 110; 53%), whilst the CD4 count test had the least frequency (*n* = 41; 22%) of repeat requests. These findings were further supported by the level of confidence clinicians reported in certain test results; clinicians had the most confidence in glucose test results (*n* = 105; 52%) and the least confidence in bacteriology (*n* = 51; 30%) test results.

**TABLE 2:** Ordering and use of laboratory results by clinicians in Malawi, October–November 2012.

Variables	All of the time n (%)	Some of the time n (%)	Never n (%)	Total N (%)
Use of requested laboratory tests	151 (70)	64 (30)	1 (0)	216 (100)
Queried laboratory results	4 (2)	189 (87)	23 (11)	216 (100)
Requested repeat tests†				
CD4 count	4 (2)	41 (22)	144 (76)	189 (100)
Malaria	4 (2)	110 (53)	94 (45)	208 (100)
Hb/FBC	4 (2)	140 (67)	66 (31)	210 (100)
Glucose	1 (1)	46 (23)	152 (76)	199 (100)
Tuberculosis microscopy	0	65 (33)	133 (67)	198 (100)
Bacteriology microscopy and culture	2 (1)	52 (26)	144(73)	198 (100)
Level of confidence in results†				
CD4 count	96 (51)	90 (48)	3 (1)	189 (100)
Malaria	74 (35)	129 (60)	11 (5)	214 (100)
Hb/FBC	73 (34)	134 (63)	6 (3)	213 (100)
Glucose	105 (52)	94 (47)	3 (1)	202 (100)
Tuberculosis microscopy	74 (36)	128 (61)	6 (3)	208 (100)
Bacteriology microscopy and culture	51 (30)	101 (60)	16 (10)	157 (100)

Hb, Haemoglobin; FBC, Full blood count.

†, Interviewed clinicians who had never ordered the test were not included in the analysis. Percentages were calculated based on the number of respondents per row.

Table 3 summarises the reasons reported by clinicians for requesting repeat tests. The main reason for ordering a repeat test was because the clinical presentation was not consistent with the laboratory test result. Other reasons reported included: doubting laboratory expertise, especially for CD4 count results (*n* = 9; 22%) and malaria results (*n* = 20; 18%). Glucose (*n* = 9; 24%) and tuberculosis microscopy (*n* = 12; 23%) tests had the highest proportion of clinicians who indicated that they requested repeat tests just to check the quality of the laboratory results.

**TABLE 3:** Reasons given by clinicians in Malawi for repeating tests, October-November 2012.

Reasons†	CD4 count n (%)	Malaria n (%)	Hb/FBC n (%)	Glucose n (%)	Tuberculosis microscopy n (%)	Bacteriology n (%)
Clinical presentation not consistent with laboratory results	13 (32)	70 (65)	79 (62)	14(38)	26 (51)	22 (43)
Monitoring	13 (32)	10 (9)	20 (16)	11(30)	8 (16)	9 (18)
Querying initial results/doubt laboratory expertise	9 (22)	20 (18)	9 (7)	3 (8)	5 (10)	7 (14)
Checking laboratory reliability	5 (12)	9 (8)	18 (14)	9 (24)	12 (23)	4 (8)
First results not well documented	1 (2)	1 (1)	1 (1)	0	0	6 (12)
Sample collection problems	0	0	0	0	0	3 (6)

Hb, Haemoglobin; FBC, Full blood count.

†, Interviewed clinicians who had never ordered the test were not included in the analysis.

### Factors associated with use of laboratory tests

Table 4 summarises the clinicians’ responses to questions about factors that influenced use of laboratory tests and situations in which they did not use results from the six laboratory tests investigated. Reasons varied by the type of test. The most common reasons across the tests were unavailability of reagents and the perception that history taking was more important than laboratory tests.

**TABLE 4:** Scenarios in which clinicians in Malawi did not use laboratory test results, October-November 2012.

Type of test	When lab tests results were not used
CD4 count	Reagents are often not available; hence, patients managed without laboratory test results History taking is more important than laboratory test results If clinical presentation is strongly suggestive of advanced disease despite laboratory results
Malaria	In cases of severe malaria If clinical symptoms suggest malaria regardless of negative malaria parasites Results came too late, after patients had been discharged Reagents are often not available; hence, patients managed without laboratory test results History taking is more important than laboratory results
Hb/FBC	Laboratory results are inconsistent with clinical presentation Reagents are often not available; hence, patients managed without laboratory test results History taking is more important than laboratory results Results came too late, after patients had been discharged Doubt laboratory expertise
Glucose	Reagents are often not available; hence, patients managed without laboratory test results History taking is more important than laboratory results Use of clinical symptoms only
Tuberculosis microscopy	Reagents are often not available; hence, patients managed without laboratory test results History taking is more important than laboratory results Results came too late, after patients had been discharged Use of experience with clinical symptoms
Bacteriology microscopy culture and sensitivity	Reagents are often not available; hence, patients managed without laboratory test results History taking is more important than laboratory results Laboratory results are not consistent with clinical presentation In cases of urinary tract infection, clinical symptomatic management is used regardless of laboratory results

Hb, Haemoglobin; FBC, Full blood count.

Table 5 summarises factors associated with use of laboratory test results. A higher proportion of clinicians working in central hospitals responded that they used laboratory test results all of the time compared with clinicians working in CHAM or district hospitals. Medical doctors were more likely to use laboratory test results all of the time. In addition, clinicians who never queried laboratory results, clinicians who interacted with the laboratory some of the time and clinicians who noted gaps in infrastructure were more likely to use the laboratory results all of the time. The type of facility that clinicians worked in was the only factor significantly associated with using laboratory test results all of the time (*P*< 0.001).

**TABLE 5:** Factors associated with use of laboratory results by clinicians in Malawi, October-November 2012.

Variable	Category	Use of lab results all the times (%)	P-value†
Type of facility	Central hospital	86/100 (86)	< 0.001
	CHAM hospital	16/28 (57)	
	District hospital	49/88 (56)	
Professional title	Medical doctor	17/21 (81)	0.692
	Clinical officer	93/133 (70)	
	Medical assistant	30/45 (67)	
	Other	11/17 (65)	
Queried laboratory results	Never	16/23 (70)	0.199
	All of the time	3/4 (75)	
	Some of the time	132/189 (70)	
Interaction with laboratory‡	Some of the time	51/66 (77)	0.313
	All of the time	92/135 (68)	
	Very limited	8/13 (62)	
Need for infrastructure§	No	13/18 (72)	0.262
	Yes	133/190 (70)	

CHAM, Christian Health Association of Malawi.

†, From the chi-squared test. *P* < 0.05 considered to be statistically significant; ‡, Two entries were missing, §One entry was ‘not sure’ and seven entries were missing.

### Laboratory interaction and desired improvements

A total of 201 (93%) clinicians reported interacting with laboratory staff either all of the time (*n* = 135; 63%) or some of the time (*n* = 66; 30%) (Table 6). Reasons for interacting with laboratory staff varied and included a combination of clinic meetings, consultation visits and being friends with laboratory personnel (data not shown).

**TABLE 6:** Laboratory interactions and desired improvements, in Malawi, October-November 2012.

Variables	n (%)
**Interaction with laboratory staff (N= 216)**	
All of the time	135 (63)
Some of the time	66 (30)
Very limited interaction	12 (6)
Never	1 (0)
Missing	2 (1)
**Infrastructural improvements† (n= 190,91%)**	
Additional equipment	139 (53)
Service of equipment	20 (8)
Expansion of laboratory	93 (35)
Improvement of laboratory	11 (4)
**Laboratory services improvements† (n= 160,77%)**	
Back up services	10 (4)
Turn-around time	63 (22)
Laboratory-clinician communication	24 (8)
Provide reference values and interpretation	10 (4)
Availability of reagents and supplies	55 (19)
Additional tests	47 (16)
Provide 24-hr services	4 (1)
Additional personnel	41 (14)
Additional qualified personnel	13 (4)
Provide reliable/quality results	10 (4)
Motivate staff	11 (4)
Stop task shifting	1 (0)
Encourage task shifting	1 (0)

†, Some clinicians had multiple laboratory infrastructural and service improvement suggestions.

A total of 190 clinicians (91%) reported that the laboratories at their facilities required infrastructural improvements. Specifically, 53% (*n* = 139) indicated that additional equipment was required and 35% (*n* = 93) said that the laboratory in their health facility should be expanded. The most frequently mentioned area of needed improvement was shorter turn-around times for test results (*n* = 63; 22%), followed by the need for consistent availability of reagents and supplies (*n* = 55; 19%) to ensure that laboratory services were uninterrupted. A small proportion indicated the need for laboratories to offer tests in addition to those currently available (*n* = 47; 16%). Whereas some clinicians indicated need for additional laboratory personnel (*n* = 41; 14%), only 4% (*n* = 13) indicated the need for qualified, skilled or specialist laboratory personnel.

### Clinician observations

A total of 97 clinician observations were completed, during which 80 (83%) tests were ordered, mainly for patients in the Medical, Outpatient Department and Paediatric wards (Table 7). All ordered tests were returned to the original wards. The final diagnosis and patient management were compared to the laboratory test results and 73 (91%) were noted as having been used by the clinicians in making a diagnosis or planning patient management. All laboratory tests ordered in the Paediatric and Surgical wards were used by the clinicians in making a diagnosis or planning patient management, whereas clinicians in other wards did not use all results.

**TABLE 7:** Clinician observations, Malawi, October-November 2012.

Ward observed	Number of observations	Test(s) ordered n (%)	Test results used n (%)
Maternity Ward	10	8/10 (80)	6/8 (75)
Medical Ward	27	23/27(85)	21/23 (91)
Outpatient Department	19	17/19 (90)	15/17 (88)
Paediatric Ward	20	15/20 (75)	15/15 (100)
Private Ward	4	4/4 (100)	3/4 (75)
Surgical Ward	16	13/16 (81)	13/13 (100)
Not indicated	1	0	0
**Total**	**97**	**80/97 (83)**	**73/80 (91)**

### Key informant interviews

Twelve key informants were interviewed (Table 8). The key informants reported that laboratory services were very useful and that the quality was good, but services were often unavailable and hence underutilised. Key recommendations were to strengthen laboratory services, supply chain and equipment maintenance services to ensure uninterrupted services.

**TABLE 8:** Key informants’ emerging themes, Malawi, October-November 2012.†

Emerging theme	Frequency	%
Laboratory services should be strengthened to offer more tests	11	36
Strengthen reagent supply chain and equipment service	6	19
Expand test menu according to need	3	10
Improve laboratory human resources and expertise	2	7
Strengthen national quality assurance and improve quality	4	13
Improve laboratory-clinician interaction	1	3
Improve laboratory utilisation by clinicians	2	6
Improve test results turn-around time	2	6
**Total**	**31**	**100**

†, Analyses based on interviews with 12 management-level individuals recruited from four MoH departments.

## Discussion

In this study, it was observed that 9 in 10 laboratory tests were being used to inform patient management decisions and that 7 in 10 clinicians reported always using the results of requested laboratory tests in patient management. This is higher than previously reported in Malawi by Mepham et al. in 2002, where 64% of clinicians were using laboratory results to inform clinical decisions.^10^ One possible explanation for the differences observed in these two studies might be attributed to laboratory strengthening efforts by Malawi's MoH and partners in increasing human resources and building a strong laboratory infrastructure over the past decade. It is also noteworthy that the previous study was conducted at a single health facility, whereas the current study included 22 health facilities and 216 clinicians across the country, thus increasing its representativeness.

In this study, we found that the vast majority of clinicians often questioned laboratory results, which led them to request repeat tests. Malaria and Hb/FBC tests were repeated most often; the main reason for requesting repeat tests was that the clinical presentation was not consistent with the laboratory results. A study in Ghana^12^ had similar findings on the high reliance of clinical symptoms in diagnosing malaria and found that clinicians’ perception of laboratory services was the main factor influencing use of laboratory test results. In another study,^13^ malaria and Hb tests had the least accurate results amongst surveyed laboratories. The observations in Malawi could be attributed to use of non-laboratory personnel to perform these quantitative and semi-quantitative tests in point-of-care settings, although further investigation would be required to establish such an association.

Clinicians provided scenarios in which laboratory tests were ordered but results not used in diagnosis and patient management. The main theme from the scenarios demonstrates the importance that clinicians place on obtaining a detailed patient history instead of making clinical decisions primarily based on laboratory results. The Malawi Standard Treatment Guidelines 2009 states that ‘[*g*]ood therapeutics depends on accurate diagnosis, based on thorough history-taking, necessary careful physical examination and, if required, supporting laboratory testing’.^14^ These treatment guidelines allow the clinicians to determine whether laboratory testing is required. This finding is similar to Petti et al.'s^15^ observation that the inadequacy of laboratory capacity to perform certain tests and assure the quality of those tests promotes the perception amongst clinicians that laboratory tests are not necessary for patient management.

Another emerging theme was the continuous unavailability of reagents, forcing clinicians to use clinical management instead of laboratory test results, even after reagents are available. A laboratory assessment conducted in 2010 across the 42 laboratories in Malawi showed that whilst central hospitals had 100% availability of reagents and consumables, district hospitals had 83% availability of reagents for HIV rapid tests, 71% for CD4 tests and 54% for haematology tests.^16^ CHAM facilities had 93% availability of reagents and consumables for rapid HIV tests, 43% for CD4 tests and 86% for haematology tests. The supply chain systems in resource-limited settings face challenges in forecasting, procurement, distribution and inventory management. In order for clinicians to use laboratory tests, these services need to be available consistently.

Clinicians who used laboratory test results all the time were more likely to work in central hospitals, be medical doctors by profession, never question laboratory test results, interact with laboratory staff some of the time, and not report the need for infrastructure improvement. However, working in a central hospital was the only factor significantly associated with the use of laboratory test results (*P*< 0.001). A national laboratory assessment conducted in 2010,^16^ found that central hospitals were well-stocked with haematology, HIV and CD4 count test reagents and consumables compared with other health facilities. Thus, consistent availability of reagents may be an influencing factor in the use of laboratory results by clinicians.

Another finding from this study was the frequent interaction of clinicians and laboratory staff, suggesting that there is a good working relationship between the two cadres. A high proportion of clinicians reported interacting with laboratory staff on a daily basis, with only 3 in 10 clinicians interacting some of the time. This high level of interaction provides an opportunity for improvements in service delivery to meet clinicians’ expectations and increase the likelihood that they will use laboratory test results in patient management. These interactions can also provide an avenue for clinicians and laboratory staff to work together to improve the laboratory-clinic interface at the time when health systems strengthening is a priority to the Malawi government and partners.

Clinicians in our study suggested infrastructural and service improvements in the laboratories at their health facilities. Additional equipment and expansion of laboratories were the most common suggestions for infrastructural improvements. The close interaction between clinicians and laboratory staff might mean that they were aware of the challenges their laboratories faced. The earlier laboratory assessment^17^ showed that only central hospitals offered laboratory tests according to a tiered test menu. Clinicians may have suggested that the need for additional equipment would address the test menu gap. In addition, turn-around time, availability of reagents and additional tests were suggested areas of needed improvement at laboratories in their health facilities. These suggestions are similar to the findings of Petti et al.^15^ that suggest barriers to effective use of laboratories in healthcare in Africa are related to the lack of laboratory consumables, essential equipment and logistical support. Availability of laboratory results in a shorter period would allow clinicians to make more comprehensive treatment decisions. Unavailability or delays in receiving laboratory results has the potential to prompt clinicians to use clinical diagnosis only versus in combination with laboratory diagnosis.

The clinician consultation observations showed that a majority of clinicians requested laboratory tests, although not all tests were used for decision making in patient management. These findings were corroborated by the clinician interviews, which indicated that whilst there are challenges in requesting laboratory tests, there are also challenges in using the results. Reasons for not using laboratory test results could be attributed to varying interpretations of Malawi's treatment guidelines, trust in laboratory quality, availability of reagents, or a laboratory's robustness in providing results within an acceptable timeframe.

The surveyed key informants’ top emerging themes were to strengthen laboratories to expand the test menu, supply chain and quality systems. The key informants’ suggestions validated the survey findings that the availability of the test menu at the health facility level influenced clinicians to use the laboratory tests. Availability of reagents would ensure consistent availability of laboratory services for clinicians. Strengthening of the laboratory quality systems would build confidence for clinicians to use the results of all laboratory tests ordered.

Availability of reagents as a factor associated with the use of laboratory test results in patient management would seem to be beyond the scope of possible interventions at the health facility level, because reagent procurement is done centrally. However, other factors, such as confidence in the test and need for better infrastructure are within the scope of interventions that could be undertaken at the health facility level. Considering that the majority of the clinicians who participated in this survey were stationed in central or referral hospitals where laboratories offered an expanded test menu, these findings provide a fairly accurate picture of the use of laboratory test results in situations where the required tests are offered. It would be a reasonable strategy to include the value of laboratory investigations in clinicians’ pre-service training and reinforce this at the health facility level through routine advocacy by the laboratory staff.

A high proportion of clinicians suggested the need for expansion, additional human resources and expertise, as well as for additional equipment. In Malawi, a national assessment of laboratory services conducted in 2010^16^ showed that district-level laboratories had a lower proportion of qualified staff compared with central hospital laboratories (32% and 46%, respectively). A third of the district laboratories had adequate space, although microbiology and biochemistry equipment were not available in most district laboratories.^16^ These might be some of the reasons clinicians suggested the improvements they did. These gaps are currently being addressed through the National Laboratory Strategic Plan (2010–2014)^17^ and the WHO accreditation process, Strengthening Laboratory Management Toward Accreditation (SLMTA).^18^

As clinicians increase utilisation of the laboratory results, expectations will continue to rise in terms expanded test menus, shorter turn-around times, good quality and interpretation of results.^19^ In view of this, it will be necessary for laboratories in Malawi to continuously re-evaluate ways of improving the laboratory-clinic interface in order to ensure test results get to the patients efficiently and laboratory services are used more frequently in the management of patient care and treatment.

### Limitations

One limitation of this study is that the clinician consultation observations were conducted at only two hospitals. Further, the presence of an observer might have influenced clinicians’ behaviour with regard to laboratory test requests and use of test results. The use of a single observer per site might have introduced some degree of subjectivity. In addition, interviews relied on self-reported information and are thus subject to bias. Clinician responses may not have been an accurate representation of laboratory test request and result usage habits, as they might have been biased towards what they know they were supposed to do as opposed to what they actually do.

### Conclusion

This study highlights the patterns and possible factors and barriers related to the use of laboratory test results by clinicians in public health facilities in Malawi. It also highlights the challenges inherent in the laboratory-clinic interface in Malawi and points to opportunities for improvement. Limited test menus, longer turn-around times, poor quality, low confidence and inconsistent availability of reagents were the major gaps that were identified in the public laboratory tier system in Malawi from the clinicians’ perspective. These gaps can deter utilisation of laboratory services by clinicians in public health facilities in Malawi. A lesson learnt from this study is the need for continuous solicitation of feedback from users of laboratory services in order to improve these services. The key recommendations from this study are to strengthen the laboratory infrastructure, supply chain and quality management systems in order to restore confidence and increase utilisation of laboratory tests in patient management. A follow-up study would provide a platform for evaluating Malawi's National Laboratory Strategic Plan and impact on the use of laboratory tests by clinicians.

## References

[1] The World Bank. Malawi [page on the Internet]. n.d. [cited 2013 Jan 22]. Available from: http://data.worldbank.org/country/malawi

[2] National Statistical Office (NSO) [Malawi] & ICF Macro. Malawi demographic and health survey 2010. Calverton, Maryland: NSO & ICF Macro; 2011.

[3] National Malaria Control Programme (NMCP) [Malawi] and ICF International. Malawi: Malaria indicator survey (MIS) 2012. Lilongwe, Malawi and Calverton, Maryland, USA: NMCP and ICF International; 2012.

[4] Lawson M, Mazengera S, Nkhoma-Mbawa F, et al. Malawi essential health services campaign. For all campaign: country case study. Oxford, UK: Oxfam International; 2008.

[5] World Health Organization. Essential health packages: What are they for? What do they change? WHO Service Delivery Seminar Series. Draft Technical Brief No. 2 [document on the Internet]. c2008 [cited 2010 Oct 21]. Available from: http://www.who.int/healthsystems/topics/delivery/technical_brief_ehp.pdf

[6] Ministry of Health. Malawi Medical Laboratory Policy. Lilongwe: MoH; 2007.

[7] Price CP. Evidence-based laboratory medicine: supporting decision-making. Clin Chem. 2000;46(8):1041–1050. 10926881

[8] Wians FH. Clinical laboratory tests: which, why, and what do the results mean? LabMedicine. 2009;40(2):105–113.

[9] Sturm AW. Rational use of antimicrobial agents and diagnostic microbiology facilities. J Antimicrob Chemother. 1988;22(2):257–260. 10.1093/jac/22.2.257 3182419

[10] Mepham SO, Squire SB, Chisuwo L, et al. Utilisation of laboratory services by health workers in a district hospital in Malawi. J Clin Pathol. 2009;62(10):935–938. 10.1136/jcp.2009.069062 19783724

[11] Mengo, D. Evidence based laboratory medicine: do doctors use laboratory results prior treatment of patients? Poster session presented at: 29th World Congress of Biomedical Laboratory Science, June 6–10, Nairobi, Kenya; 2010.

[12] Polage CR, Bedu-Addo G, Owusu-Ofori A, et al. Laboratory use in Ghana: physician perception and practice. Am J Trop Med Hyg. 2006;75(3):526–531. 16968935

[13] Bates I, Bekoe V & Asamoa-Adu A. Improving the accuracy of malaria-related laboratory tests in Ghana. 2004;3:38. 10.1186/1475-2875-3-38 PMC529273 15516269

[14] Ministry of Health. Malawi standard treatment guidelines. 4th edition. Malawi: Ministry of Health; 2009.

[15] Petti CA, Polage CR, Quinn TC, et al. Laboratory medicine in Africa: a barrier to effective health care. Clin Infect Dis. 2006;42(3):377–382. 10.1086/499363 16392084

[16] Ministry of Health. National laboratory assessment: baseline report. Malawi: Ministry of Health; 2010 (Unpublished report).

[17] Ministry of Health. 5-year national laboratory strategic plan. Malawi: Ministry of Health; 2010

[18] Gershy-Damet GM, Rotz P, Cross D, et al. The World Health Organization African region laboratory accreditation process: improving the quality of laboratory systems in the African region. Am J Clin Pathol. 2010;134(3):393–400. 10.1309/AJCPTUUC2V1WJQBM 20716795

[19] Dubois C, Swartz V. A vision for change: the expanding role of clinical diagnosis is evident. Information Technology Series [page on the Internet]. c2011 [cited 2011 Nov 12]. Available from: http://laboratory-manager.advancedweb.com/Archives/Article-Archives/A-Vision-for-Change.aspx

